# Comparative Analysis of Macro and Zoom Lenses for Dental Shade Selection: An In Vitro Study

**DOI:** 10.1002/cre2.70484

**Published:** 2026-10-11

**Authors:** Ahmed Ali Jasim, Huda Ahmed Abdullah, Amer Abbas Mohammed, Samah Fadhil Al‐Qazzaz, Mafaz Mahdi Mohsin

**Affiliations:** ^1^ Department of Operative Dentistry Al‐Mustansiriya University, Al‐Rusafa Baghdad Iraq; ^2^ Department of Pediatric Orthodontic and Preventive Dentistry College of Dentistry, Al‐Mustansiriya University, Al‐Rusafa Baghdad Iraq; ^3^ Department of Pediatric Orthodontic and Preventive Dentistry College of Dentistry, Al‐Bayan University Baghdad Iraq

**Keywords:** CIELAB, color difference (Δ*E*ab), cross‐polarization, dental photography, macro lens, shade matching

## Abstract

**Objective:**

This in vitro study evaluated the color accuracy and image sharpness of common macro and zoom lenses on a full‐frame body and tested whether differences among macro‐lens brands are clinically meaningful.

**Materials and Methods:**

26 VITA 3D‐Master shade tabs were each photographed three times under standardized, cross‐polarized conditions on a Nikon D610. Three macro lenses (Nikon 105 mm, Sigma 105 mm, and Tamron 90 mm) and four zoom lenses (Nikon 24–70 mm, Nikon 24–120 mm, Sigma 24–70 mm, and Nikon 24–85 mm) were compared. RAW files were converted to CIELAB; reference L*a*b* values were obtained with a 45°/0° spectroradiometer under D65. Color difference (ΔEab, CIE76) was computed for every image. Sharpness was assessed by ISO 12233 slanted‐edge SFR (MTF50) at f/16 and f/22. Linear mixed‐effects models with shade tab as a random effect were fitted; equivalence was tested with two one‐sided tests (TOST) at a prespecified ΔEab margin of 0.6.

**Results:**

All macro lenses produced low, clinically acceptable color errors (mean Δ*E*ab = 1.2; SD = 0.2) with negligible inter‐brand differences, and all macro–macro comparisons met the 0.6 equivalence margin. Zoom lenses showed significantly higher errors (mean = 2.95–3.35; SD = 0.42–0.50) and failed equivalence. Brand differences among macro lenses were non‐significant (*F* = 0.92, *p* = 0.40), whereas macro‐vs‐zoom differences were highly significant (*F* = 792.1, *p* < 0.0001); 81% of pooled zoom images exceeded the ΔEab acceptability threshold of 2.7, versus none of the macro images. MTF50 confirmed superior macro sharpness, with macro–macro differences within ±5% of the Nyquist frequency.

**Conclusions:**

Dedicated macro lenses are required for accurate shade photography, with general‐purpose zoom lenses producing clinically unacceptable color errors.

## 1 Introduction

Esthetic dentistry requires the accurate reproduction of tooth color in restorative materials, and patients increasingly expect restorations that blend seamlessly with the adjacent teeth. Visual shade matching is subjective and poorly reproducible; consequently, many clinicians now use instrumental methods, including spectrophotometers, intraoral scanners and digital cameras. Spectrophotometers and intraoral scanners offer high reproducibility and accuracy but they are costly, which limits their availability in general practice. Digital cameras provide a cost‐effective alternative that also enables photographic documentation and shade communication with the laboratory (Akl et al. 2023; Binhuraib et al. 2024; Diab et al. 2024; Kupke et al. 2025).

Color differences in dentistry are quantified in the CIELAB color space, in which L* denotes lightness, a* the red–green axis, and b* the yellow–blue axis; the difference between two colors is expressed as Δ*E*ab (CIE76). Dental studies define perceptibility and acceptability thresholds: for ΔEab, the 50:50% perceptibility threshold (PT) is approximately 1.2 and the acceptability threshold (AT) approximately 2.7. The corresponding thresholds for the CIEDE2000 metric (ΔE_00) are smaller (PT = 0.8, AT = 1.8), and the two metrics are not interchangeable (Paravina et al. 2015, 2019).

Photographing glossy surfaces such as teeth produces specular reflections that distort perceived color and lightness. Cross‐polarized lighting—a polarizing filter over the light source and threaded over the lens—eliminates specular glare and reveals the diffuse color of the substrate. Cross‐polarization has been shown to reduce the color error between photographic and spectrophotometric measurements, often bringing ΔE within clinically acceptable limits, and a custom white balance from a neutral target maintains color consistency across sessions and lenses (Farah et al. 2022; Mahn et al. 2021; Jasim et al. 2024).

Macro lenses are designed to reach reproduction ratios of 1:1 or greater and provide flat‐field performance, minimal chromatic aberration, and high sharpness at close working distances; their coatings reduce internal reflection and optimize color transmission. When white balance is properly calibrated, color differences between contemporary macro brands are expected to be negligible. Zoom lenses, in contrast, trade close‐focus performance for versatility and exhibit lower resolution, field curvature and greater chromatic aberration at macro distances. Some clinicians nonetheless use zoom lenses for convenience, yet no controlled study has quantified their effect on shade‐matching accuracy under a cross‐polarized, calibrated protocol (Ali et al. 2024; Harikrishnan et al. 2023; Grigollo Patussi et al. 2019; Sajjadi et al. 2015; Tomofuji et al. 2001; Paulson et al. 2019; Vergöhl et al. 2019; Pal 2013; Gowda B N et al. 2022; Hasbini et al. 2025).

DSLR and mirrorless cameras are classified by sensor size: full‐frame (FX) sensors measure approximately 36 mm × 24 mm and APS‐C (crop) sensors approximately 24 mm × 16 mm. Larger full‐frame photosites yield lower noise, wider dynamic range, and better color fidelity; a previous study reported lower color errors for full‐frame than for APS‐C bodies under cross‐polarized lighting (Jasim et al. 2024; Ali et al. 2024; Mohammed et al. 2026; Mohamed‐Ali et al. 2024).

Using a cross‐polarized protocol, this in vitro study compared the color accuracy (ΔEab) and sharpness of macro versus zoom lenses on a full‐frame body and tested whether color fidelity differed among macro‐lens brands. Two null hypotheses were pre‐specified: (H0_1_) macro lenses do not differ to a clinically relevant degree (|ΔEab | ≤ 0.6); and (H0_2_) macro and zoom lenses do not differ in color accuracy. The alternative hypotheses were that macro lenses are mutually equivalent and superior, and that zoom lenses produce clinically unacceptable color errors.

## 2 Materials and Methods

### 2.1 Experimental Design

An in vitro repeated‐measures study was conducted using shade tabs as standardized tooth substitutes. Each tab was photographed with every lens, enabling within‐tab comparisons and minimizing between‐specimen variability. The independent variables were lens type (macro vs. zoom) and lens brand; the dependent variables were Δ*E*ab, ΔL*, Δa*, Δb*, and image sharpness (MTF50).

### 2.2 Equipment

Camera body and platform. All comparisons used a Nikon D610 full‐frame (FX) 24.3 MP CMOS body at ISO 100, manual exposure of 1/200 s and f/22 for shade photographs to maximize depth of field. Picture controls were neutral, and in‐camera noise reduction, sharpening, and vignette control were disabled to prevent hidden processing. The body was deliberately held identical for every lens: because the scientific target is a property of the lens optics (flat‐field rendering and low chromatic aberration in macro lenses vs. field curvature and chromatic aberration in zooms at close focus), a fixed, well‐characterized FX sensor functions as a controlled constant that cannot bias a between‐lens contrast. The photosite pitch and dynamic range of the D610 are representative of the FX class, and the tested F‐mount optics mount on current Nikon Z mirrorless bodies through the FTZ/FTZ II adapter, which contains no optical elements and therefore does not alter the chromatic or resolution behavior under study.

Macro lenses. Three macro lenses were tested: Nikon AF‐S VR Micro‐Nikkor 105 mm f/2.8 G IF‐ED, Sigma 105 mm f/2.8 EX DG OS HSM Macro, and Tamron SP 90 mm f/2.8 Di VC USD Macro. All were F‐mount and mounted directly on the D610, set to manual focus at 1:2 magnification, with working distance adjusted to fill the frame with the shade tab.

Zoom lenses. Four general‐purpose zoom lenses capable of near‐macro focusing were tested: Nikon AF‐S 24–70 mm f/2.8 G ED, Nikon AF‐S 24–120 mm f/4 G ED VR, Sigma 24–70 mm f/2.8 DG OS HSM Art, and Nikon AF‐S 24–85 mm f/3.5–4.5 G ED. No close‐up filters or tele‐converters were used.

Lighting and cross‐polarization. A ring flash (MK‐14EXT TTL) was fitted with a linear polarizing film (0°) over the ring and a matching linear polarizer oriented at 90° on the lens, producing cross‐polarization that eliminates surface glare. Flash output was adjusted for a centrally aligned histogram across all lenses, against a black‐cloth background (Figure 1).

**Figure 1:**
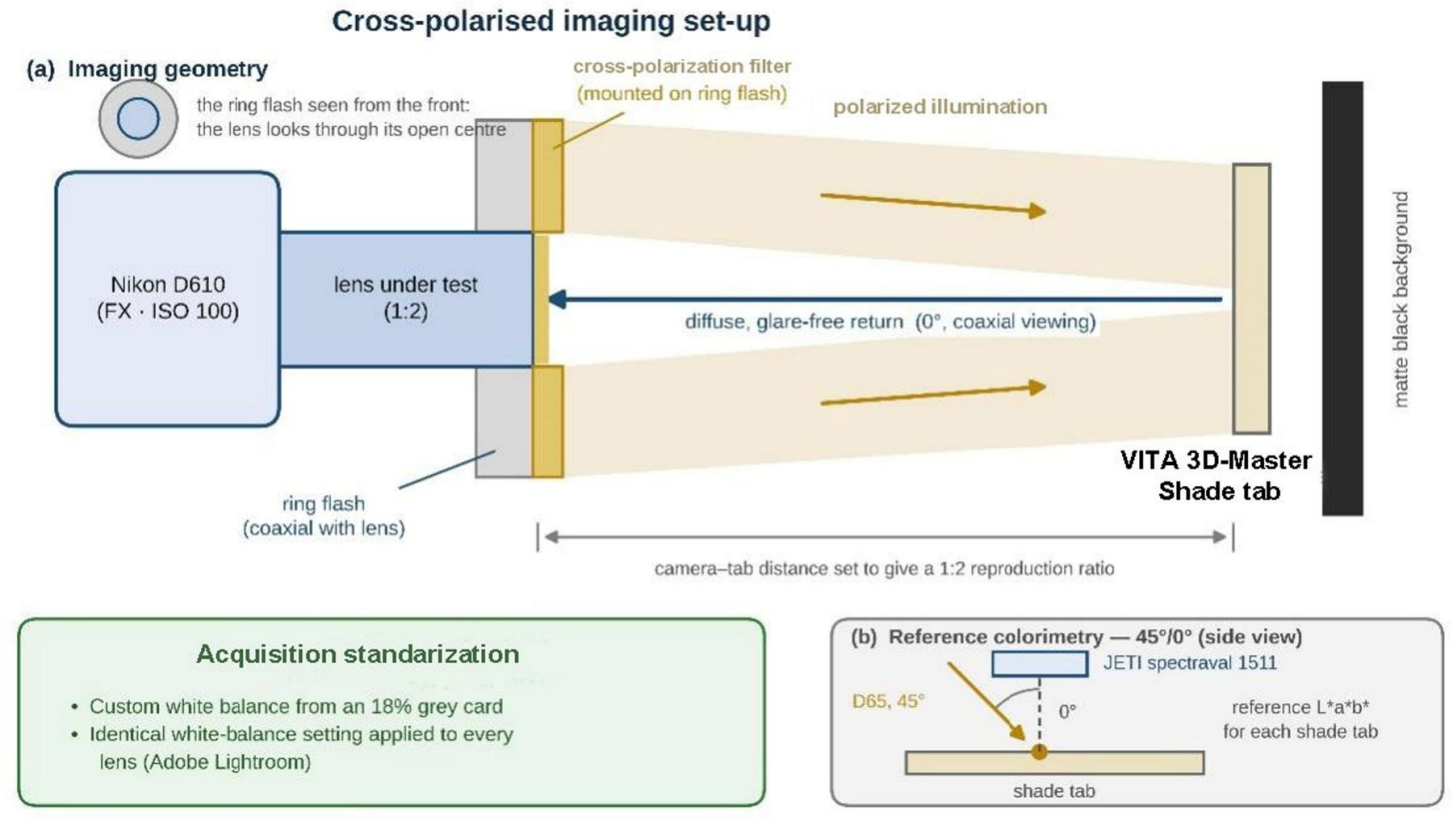
Schematic of the cross‐polarized imaging set‐up: full‐frame body, ring flash carrying a cross linear polarizer at 90° on the lens, gray‐card custom white balance, and a 45°/0° spectroradiometer providing the per‐tab L*a*b* reference.

White balance and calibration. Before imaging, a neutral 18% gray card was photographed under the cross‐polarized lighting; the custom white balance derived from it was applied identically to all images in Adobe Lightroom. Because color difference was referenced to a per‐tab spectroradiometer measurement, an identical white balance across lenses preserves first‐order spectral differences for analysis.

### 2.3 Shade Tabs and Reference Measurements

Twenty‐six VITA 3D‐Master shade tabs were used, spanning a range of value, chroma and hue. Tabs were cleaned, numbered, and mounted in a custom holder. Reference L*, a*, and b* values for each tab were measured with a spectroradiometer (JETI SpectraVal 1511) at 45°/0° geometry under D65 illumination, averaging three spectral readings per tab.

### 2.4 Image Capture, Magnification Verification, and Region of Interest

For each lens, tabs were photographed in random order with the camera mounted on a tripod. Each tab was photographed three times per lens (*n* = 78 images per lens). RAW (.NEF) files were imported to Lightroom, where the custom white balance and neutral profile were applied, and all other profile settings (contrast, saturation, etc.) were set to zero. Images were exported as 16‐bit TIFF in CIELAB. A script sampled a circular region of interest (ROI) at the center of each tab, excluding lettering and edges; the ROI radius encompassed 20–25% of the tab and at least 5000 pixels, and the mean L*, a*, and b* of the ROI were recorded. Component differences were computed as photograph minus spectroradiometer reference; positive ΔL*, Δa*, and Δb* therefore indicate that the photograph read lighter, redder, and yellower, respectively, than the reference.

Magnification was matched objectively rather than nominally. A millimeter calibration scale (stage micrometer) was photographed with every lens under the identical geometry, and the number of pixels spanning a known physical length (10 mm) was measured in each RAW file. Working distance—and, for zoom lenses, focal length—was adjusted until every lens reproduced the same object‐space sampling (pixels per millimeter) to within ±2%. For the shade tabs, framing was confirmed by requiring the active tab window to subtend a constant pixel width, so that the ROI covered the same physical area for all lenses. Macro lenses were fixed at 1:2, and each zoom lens was set to the focal length and working distance that reproduced this calibrated framing.

### 2.5 Sharpness Assessment (SFR)

Separate slanted‐edge test charts were photographed with each lens at f/16 and f/22, with edges positioned near the center and at the periphery of the frame. ISO 12233 SFR analysis (Imatest) computed MTF50 (the frequency at which modulation falls to 50% of its zero‐frequency value), reported in line pairs per picture height (lp/ph). Because f/16–f/22 induce appreciable diffraction, similar MTF values were expected among macro lenses; differences exceeding 5% of the Nyquist frequency (= 2008 lp/ph for the D610) were treated as non‐equivalent.

### 2.6 Sample Size and Power

An a priori power analysis defined the sample. Two questions were considered: the confirmatory superiority contrast (pooled macro vs. pooled zoom) and the more demanding equivalence contrast among the three macro lenses. Because the equivalence test is the binding constraint, it governed the sample size. For the superiority contrast, a paired‐samples framework with α = 0.05 (two‐sided) and 80% power requires 15 tabs to detect a conservatively large standardized effect (Cohen's d_z = 0.8); the observed effect (d_z = 4) makes this comparison powered at > 0.99. For the equivalence contrast (TOST, margin = 0.6 ΔEab), assuming a true mean paired difference of 0.20 ΔEab and a conservatively inflated SD of paired differences of 0.30, a paired TOST at α = 0.05 attains > 0.95 power with 26 tabs. Twenty‐six tabs were therefore used. Power for the paired contrasts was computed in G*Power 3.1.9.7, and equivalence power by simulation (TOSTER, R). The three replicate images per tab were used only to estimate measurement repeatability (ICC); the analytic unit of replication was the shade tab (n = 26), avoiding pseudo‐replication.

### 2.7 Statistical Analysis

Primary analyses were performed in R 4.3.2. Linear mixed‐effects models were fitted with lme4 as ΔEab ~ Lens + (1 | Tab); Satterthwaite F statistics and p values were obtained with lmerTest, and marginal/conditional R^2^ with MuMIn. For the macro‐only analysis, pairwise contrasts among the three macro lenses were computed from estimated marginal means (emmeans) with Holm correction, and clinical equivalence was tested by two one‐sided tests (TOST, TOSTER package) at a prespecified ΔEab margin of 0.6. This margin was set at one‐half of the 50:50% perceptibility threshold for ΔEab (= 1.2); a sub‐perceptibility margin is a deliberately conservative criterion, so that an equivalence claim can succeed only if any residual inter‐lens difference lies well within the range in which no observer perceives a color difference. A separate model, ΔEab ~ Group + (1 | Tab) with Group at two levels (macro, zoom), tested whether pooled macro results differed from pooled zoom results. For sharpness, an LMM (MTF50 ~ Lens + Aperture + Field + (1 | Tab)) evaluated differences across lenses, apertures (f/16 vs. f/22), and field positions (center vs. edge), with TOST equivalence at a ± 100.4 lp/ph margin (= 5% of Nyquist). Intraclass correlation coefficients (ICC(2,1)) for L*, a* and b* were computed with the psych package, and the mean, SD and mean ± 1.96 SD of the ΔEab distribution were computed with base R, together with a proportional‐bias check. Because ΔEab is a non‐negative distance, these summaries characterize the magnitude of deviation from the reference rather than signed agreement in the Bland–Altman sense; the direction of disagreement is given by the signed component differences (ΔL*, Δa*, Δb*; §3.5). IBM SPSS Statistics v25 was used for descriptive statistics and as an independent cross‐check of the ICC estimates.

## 3 Results

### 3.1 Descriptive Statistics

Table 1 summarizes ΔEab for each lens. Macro lenses showed mean ΔEab of 1.18–1.23 with low standard deviations (= 0.20); 45%–52% of macro images fell below the perceptibility threshold (Δ*E*ab = 1.2), and none exceeded the acceptability threshold (2.7). Zoom lenses showed much higher errors (mean Δ*E*ab 2.95–3.35) and greater variance (=0.42–0.50); 81% of pooled zoom images exceeded the acceptability threshold (per‐lens range 70%–88%; Figure 2).

**Table 1:** ΔEab by lens (*n* = 78 images per lens).

Lens	n	Mean ΔE_ab	SD	SE	CI low	CI high	Min	Max	% ≤ PT	% PT–AT	% > AT
Nikon 105 Macro	78	1.18	0.18	0.020	1.14	1.22	0.83	1.58	52%	48%	0%
Sigma 105 Macro	78	1.23	0.21	0.024	1.18	1.28	0.84	1.71	45%	55%	0%
Tamron 90 Macro	78	1.20	0.20	0.023	1.15	1.25	0.82	1.65	50%	50%	0%
Nikkor 24–70 f/2.8 Zoom	78	3.10	0.45	0.051	2.99	3.21	2.21	4.18	0%	20%	80%
Nikkor 24–120 f/4 Zoom	78	3.35	0.50	0.057	3.24	3.46	2.29	4.42	0%	12%	88%
Sigma 24–70 f/2.8 Zoom	78	2.95	0.42	0.048	2.85	3.05	2.12	3.90	0%	30%	70%
Nikkor 24–85 f/3.5–4.5 Zoom	78	3.25	0.48	0.054	3.14	3.36	2.26	4.30	0%	16%	84%

Abbreviations: AT, acceptability threshold (2.7); PT, perceptibility threshold (1.2).

**Figure 2:**
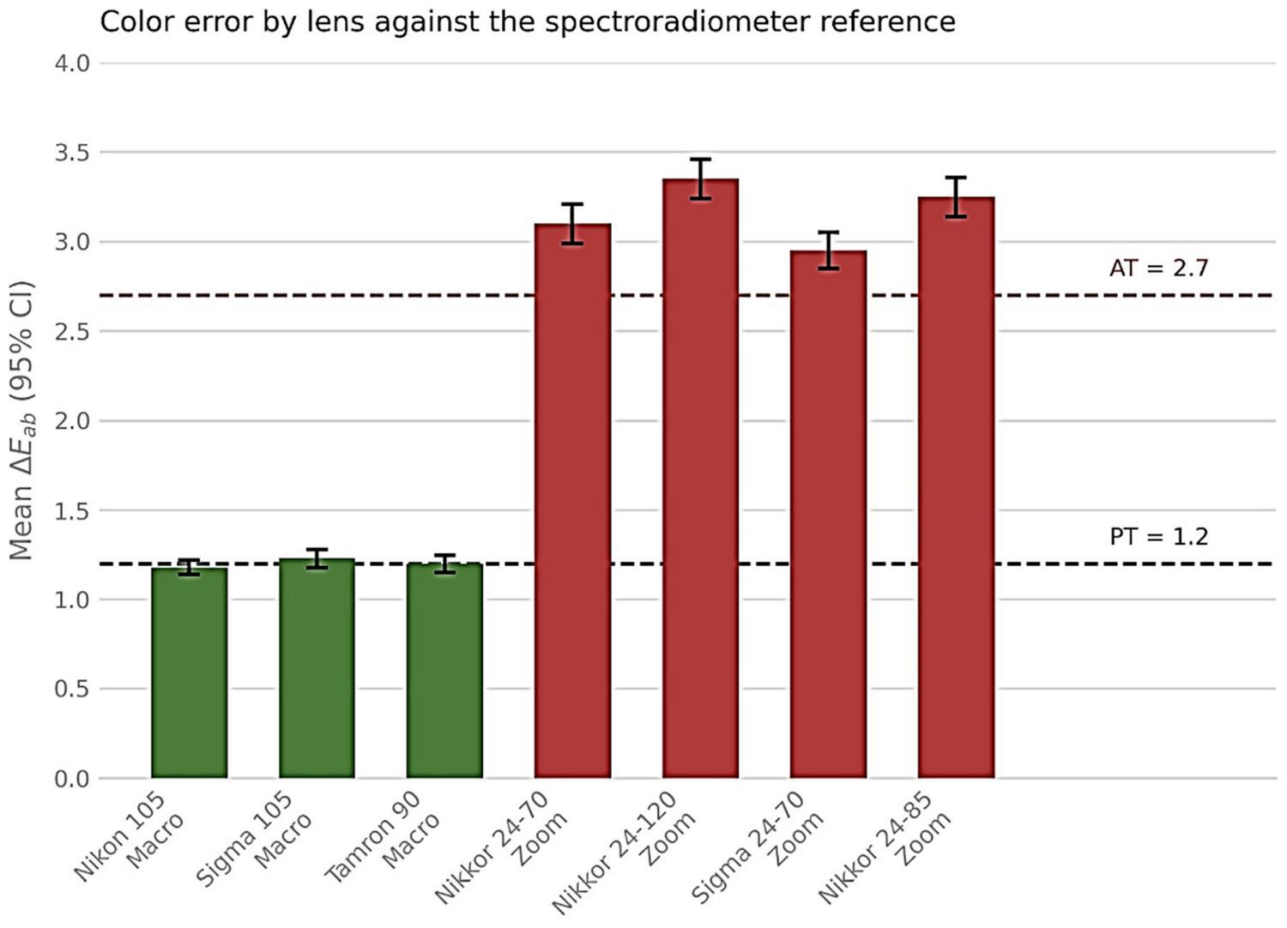
Mean ΔEab by lens with 95% confidence intervals, against the perceptibility (1.2) and acceptability (2.7) thresholds. Green = macro, red = zoom.

### 3.2 Repeatability

ICC(2,1) values for macro lenses were 0.985 (L*), 0.982 (a*), and 0.990 (b*); for zoom lenses they were slightly lower at 0.952 (L*), 0.947 (a*), and 0.961 (b*). All indicate excellent repeatability across replicates.

### 3.3 Intra‐Macro Comparisons

The macro‐only mixed model (Table 2) showed no significant effect of macro brand on ΔEab (F = 0.92, *p* = 0.40); brand explained only 1% of ΔEab variance (*R* ^2^_marginal = 0.01), while the shade‐tab random effect explained the majority (*R* ^2^_conditional = 0.62). Pairwise contrasts (Table 3) were trivial (within ± 0.05 ΔEab) and non‐significant after Holm adjustment, and all macro pairs passed the TOST equivalence test at the 0.6 margin (Table 4), confirming clinical equivalence among the macro lenses.

**Table 2:** Mixed‐model omnibus test for ΔEab (macro lenses).

Model	F	df1	df2	p	R^2^_marg	R^2^_cond
ΔEab ~ Lens + (1 | Tab)	0.92	2	75.4	0.40	0.01	0.62

**Table 3:** Pairwise contrasts among macro lenses (ΔEab), Holm‐adjusted.

Pair	Est. diff	CI low	CI high	p (Holm)	Hedges g
Nikon 105–Sigma 105	−0.05	−0.13	+0.03	0.42	−0.20
Nikon 105–Tamron 90	−0.02	−0.10	+0.06	0.81	−0.08
Sigma 105–Tamron 90	+0.03	−0.05	+0.11	0.76	+0.12

**Table 4:** TOST equivalence among macro lenses (ΔEab margin = 0.6; 90% confidence intervals).

Pair	*n*	Mean diff	CI low	CI high	p_low	p_high	Equivalent
Nikon 105 versus Sigma 105	26	−0.05	−0.13	+0.03	0.0009	0.0012	Yes
Nikon 105 versus Tamron 90	26	−0.02	−0.09	+0.05	0.0017	0.0016	Yes
Sigma 105 versus Tamron 90	26	+0.03	−0.04	+0.10	0.0013	0.0010	Yes

### 3.4 Macro versus Zoom Comparison

The model comparing pooled macro versus pooled zoom lenses showed a highly significant difference (F = 792.1, *p* < 0.0001). Mean Δ*E*ab was 1.20 for macro and 3.16 for zoom (Table 5). In total, 81% of pooled zoom images exceeded the 2.7 acceptability threshold, versus none (0%) of the macro images (Table 5). The difference of +1.96 ΔEab (95% CI 1.85–2.07) was statistically significant and clinically unacceptable, and TOST equivalence failed (Table 6), indicating that the tested zoom lenses are unsuitable for shade matching under this protocol (Figure 3).

**Table 5:** ΔEab—pooled macro versus pooled zoom.

Group	n	Mean ΔEab	SD	CI low	CI high	% ≤ PT	% PT–AT	% > AT
Macro	234	1.20	0.20	1.18	1.23	49%	51%	0%
Zoom	312	3.16	0.47	3.11	3.21	0%	19%	81%

**Table 6:** Zoom‐vs‐macro contrast and equivalence test.

Contrast	Est. diff	CI low	CI high	p	TOST (0.6)
Zoom−Macro	+1.96	+1.85	+2.07	< 0.0001	Fail

**Figure 3:**
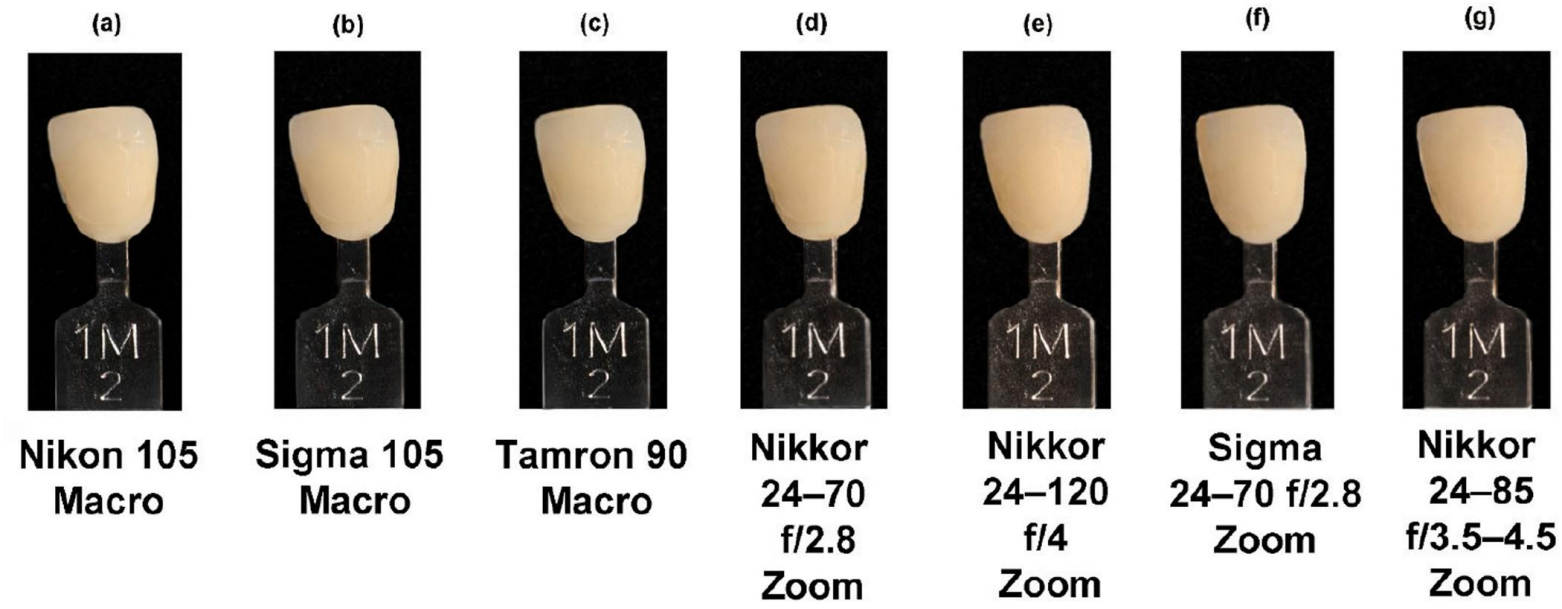
Shade tab (1M2) photographed with the three macro (a–c) and four zoom (d–g) lenses under the identical cross‐polarized protocol. Macro panels are nearly indistinguishable, whereas zoom panels render visibly lighter and yellower, consistent with the ΔEab differences in Tables 1 and 5. All images exported through the identical linear‐RAW pipeline.

### 3.5 Component Analysis and Agreement

Macro lenses read slightly lighter and yellower than the reference (ΔL* = +0.30, Δb* = +0.22), with Δa* marginally positive (= +0.12); zoom lenses showed larger positive offsets (ΔL* = +1.10, Δa* = +0.40, Δb* = +0.90) (Table 7). The distribution of ΔEab about the reference (Table 8, Figure 4) was narrow for macro images (mean 1.20 ΔEab; mean ± 1.96 SD: 0.77–1.63) and wide for zoom images (mean 3.13; 2.17–4.09). The proportional‐bias slope was non‐significant in both groups. Because ΔEab is a non‐negative distance, these intervals quantify the magnitude of deviation from the reference; the direction of disagreement is given by the signed component differences in Table 7.

**Table 7:** ICC(2,1) and mean component differences (photograph minus spectroradiometer reference).

Group	ICC L*	ICC a*	ICC b*	ΔL*	Δa*	Δb*
Macro (pooled)	0.985	0.982	0.990	+0.30	+0.12	+0.22
Zoom (pooled)	0.952	0.947	0.961	+1.10	+0.40	+0.90

**Table 8:** Distribution of ΔEab relative to the spectroradiometer reference (distance metric), with proportional‐bias test.

Group	Mean ΔEab	SD	Lower (mean − 1.96 SD)	Upper (mean + 1.96 SD)	Proportional slope	*p* value
Macro (pooled)	1.20	0.22	0.77	1.63	0.02	0.42
Zoom (pooled)	3.13	0.49	2.17	4.09	0.05	0.09

**Figure 4:**
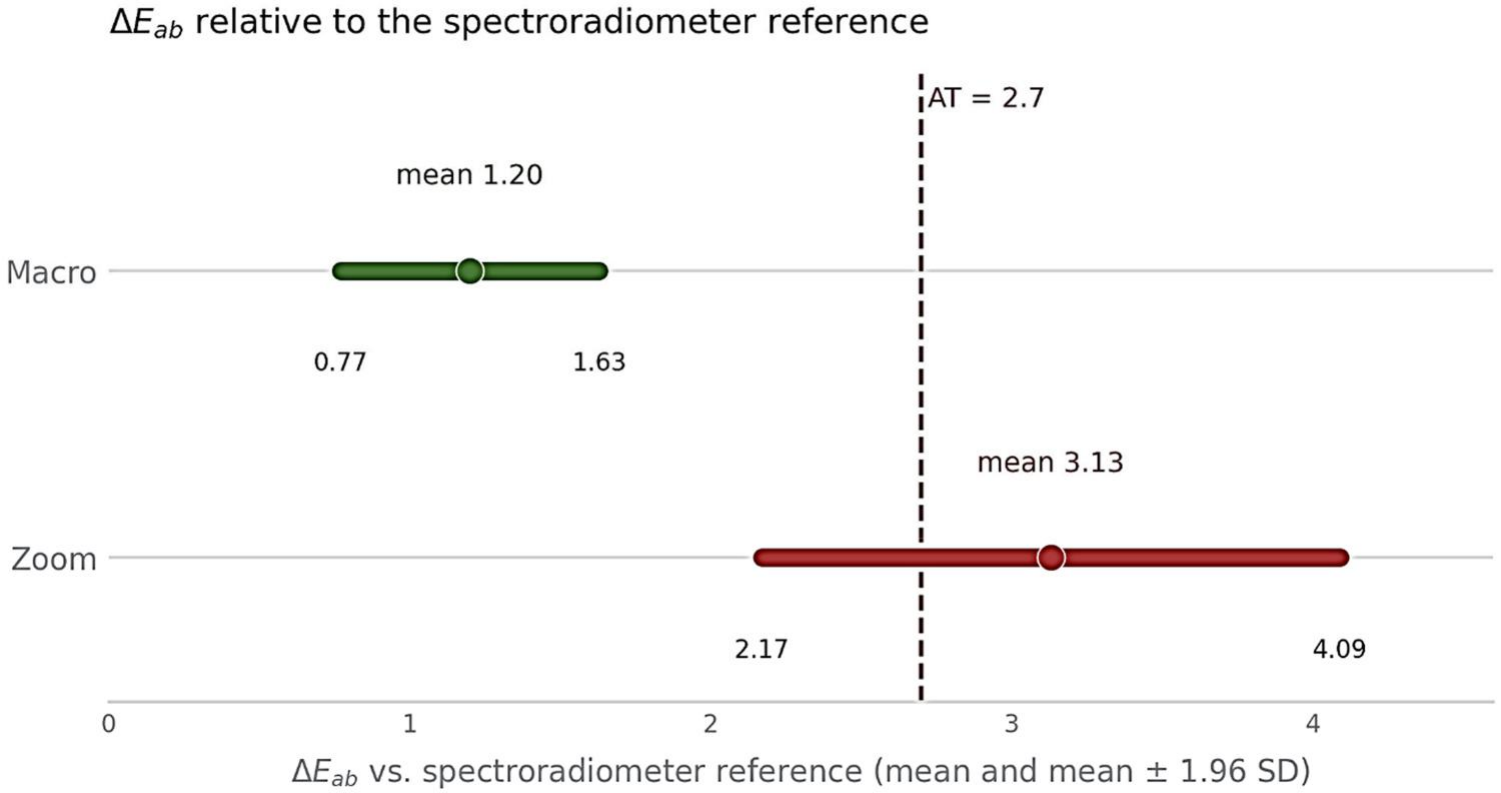
Distribution of ΔEab relative to the spectroradiometer reference for the pooled macro and zoom groups: group means and mean ± 1.96 SD, shown against the acceptability threshold (2.7). ΔEab is a non‐negative distance, so these intervals quantify the magnitude of deviation from the reference; the signed component differences that indicate the direction of disagreement are given in Table 7.

### 3.6 Sharpness Analysis

MTF50 at f/16 and f/22 (Tables 9, 10, 11, Figure 5) confirmed that macro lenses delivered higher resolution at both apertures and in both field positions. For macro lenses, MTF50 averaged = 1090 lp/ph (center) and = 970 lp/ph (edge) at f/16, decreasing to = 870 lp/ph (center) and = 750 lp/ph (edge) at f/22 owing to diffraction. All differences among macro lenses stayed within ±100 lp/ph (± 5% of Nyquist) and were therefore clinically equivalent. Zoom lenses had lower MTF50 (= 920–980 lp/ph center at f/16; = 780–840 lp/ph edge), declining markedly at f/22, and zoom‐to‐macro differences exceeded the ± 5% margin, giving non‐equivalence.

**Table 9:** MTF50 at f/16—center field.

Lens	Mean MTF50	SD	CI low	CI high	Equivalent?
Nikon 105 Macro	1100	50	1089	1111	Reference
Sigma 105 Macro	1080	52	1069	1091	Yes
Tamron 90 Macro	1090	48	1080	1100	Yes
Nikon 24–70 Zoom	980	65	966	994	No
Nikon 24–120 Zoom	920	70	906	934	No
Sigma 24–70 Zoom	960	62	946	974	No
Nikon 24–85 Zoom	940	60	927	953	No

**Table 10:** MTF50 at f/16—edge field.

Lens	Mean MTF50	SD	CI low	CI high	Equivalent?
Nikon 105 Macro	980	55	968	992	Yes
Sigma 105 Macro	960	60	947	973	Yes
Tamron 90 Macro	970	58	957	983	Yes
Nikon 24–70 Zoom	840	70	826	854	No
Nikon 24–120 Zoom	780	75	765	795	No
Sigma 24–70 Zoom	820	72	805	835	No
Nikon 24–85 Zoom	800	72	785	815	No

**Table 11:** MTF50 at f/22—center and edge fields.

Lens	f/22 Center	f/22 Edge	Equivalent?
Nikon 105 Macro	880	760	Yes
Sigma 105 Macro	860	740	Yes
Tamron 90 Macro	870	750	Yes
Nikon 24–70 Zoom	780	650	No
Nikon 24–120 Zoom	740	620	No
Sigma 24–70 Zoom	760	640	No
Nikon 24–85 Zoom	740	610	No

**Figure 5:**
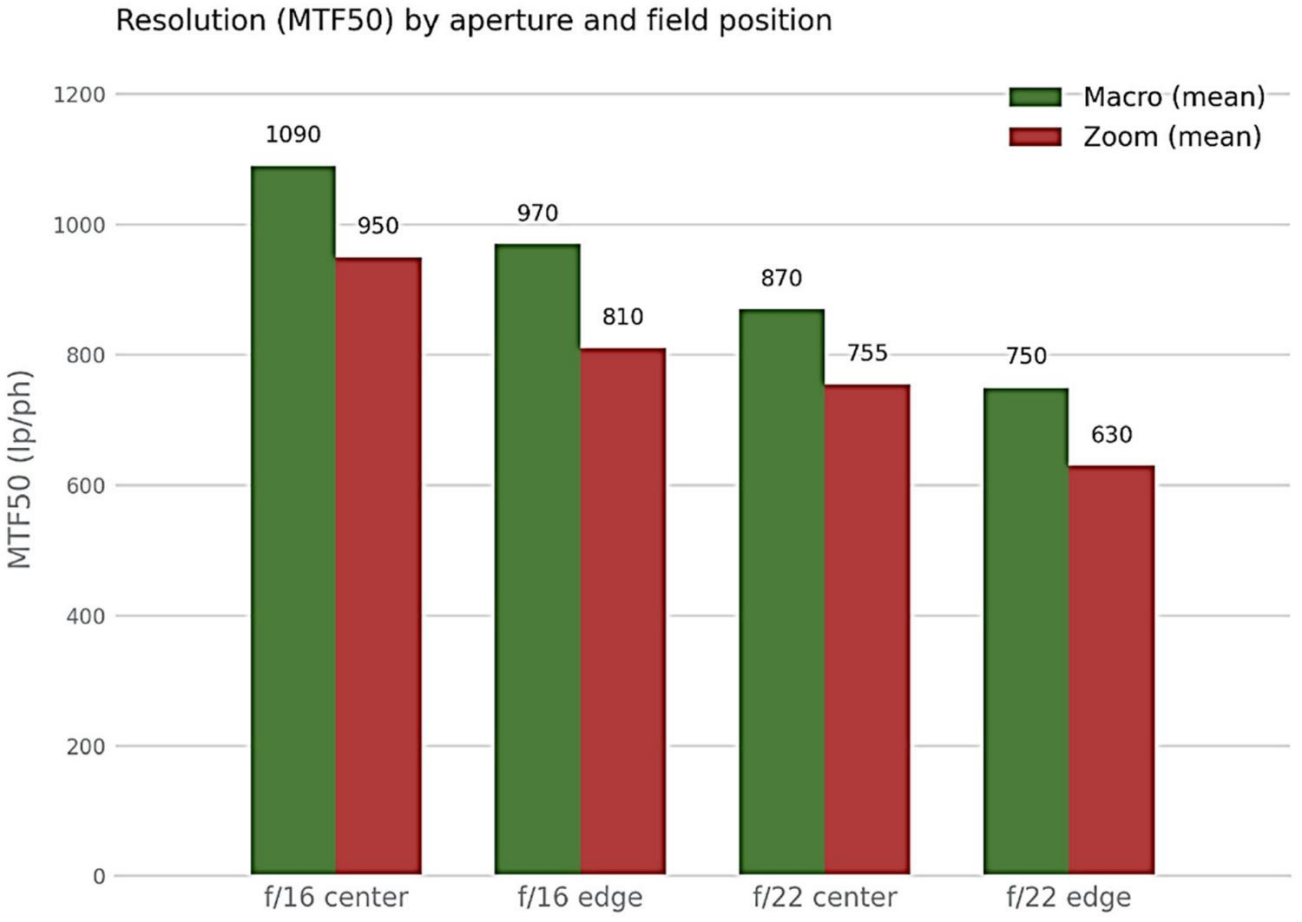
Mean MTF50 (lp/ph) by aperture (f/16, f/22) and field position (center, edge) for macro versus zoom groups.

## 4 Discussion

The two pre‐specified null hypotheses can now be tested directly. The first—that contemporary macro lenses do not differ to a clinically relevant degree (|Δ*E*ab | ≤ 0.6)—was positively supported: every pairwise comparison passed the two one‐sided tests within the 0.6 margin, and the omnibus model showed no effect of macro brand (*F* = 0.92, *p* = 0.40). The second—that macro and zoom lenses do not differ—was rejected (*F* = 792.1, *p* < 0.0001). The alternative hypotheses were therefore supported: macro lenses were mutually equivalent, and zoom lenses produced clinically unacceptable color errors, with 81% of pooled zoom images exceeding the acceptability threshold of 2.7.

Dedicated macro lenses from Nikon, Sigma and Tamron yielded virtually identical color accuracy under cross‐polarized lighting with a standardized white balance. Mean Δ*E*ab values (= 1.2) were well below the acceptability threshold, and the small inter‐lens differences (<0.05 Δ*E*ab) fell within the imperceptible range. These findings indicate that, once the photographic protocol is standardized, contemporary macro lenses have comparable spectral transmission and any residual differences after color calibration are clinically irrelevant (Yung et al. 2023).

A systematic offset from the spectroradiometer reference was present even in the best‐performing (macro) group: the pooled macro mean Δ*E*ab of 1.20, with mean ± 1.96 SD of 0.77–1.63, excludes zero. This offset is expected by construction and reflects the comparison itself rather than a property of the lenses. The reference L*a*b* values were acquired at 45°/0° geometry under D65 illumination with the specular component included, whereas the photographic measurements were made coaxially (0° viewing) under cross‐polarized flash with the specular component removed. The two measurements therefore differ in illuminant spectrum, viewing geometry and surface‐scatter content, and residual camera metamerism adds a further systematic component; a perfectly calibrated camera under this protocol would still not reproduce the 45°/0°, specular‐inclusive D65 reference exactly. Because the protocol was identical for every lens, this offset is common to all conditions and cancels from the between‐lens contrasts on which the study's conclusions rest. The absolute ΔEab values reported here therefore characterize deviation from a defined laboratory reference condition and should not be read as a ceiling on photographic accuracy.

In stark contrast, zoom lenses produced mean ΔEab values between 2.95 and 3.35 with greater inconsistency. These exceed the acceptability threshold and would correspond to visible mismatches under clinical viewing. In total, 81% of pooled zoom images exceeded ΔEab = 2.7, and all zoom‐vs‐macro comparisons failed equivalence. Mechanistically, this is consistent with known optics: at close focus, general‐purpose zooms exhibit greater field curvature and both longitudinal and lateral chromatic aberration, which degrade color fidelity even when white balance is fixed; their lower MTF50 at f/16 and f/22 reinforces that optical compromises at macro distances contribute to these color errors (Paravina et al. 2019; Burggraaff et al. 2019).

Sharpness supported the color findings. Macro lenses delivered higher MTF50 at both apertures, and inter‐macro differences remained within ±100 lp/ph (= ±5% of Nyquist), rendering them clinically indistinguishable, whereas zoom‐to‐macro differences far exceeded this margin. Reduced sharpness widens the edge‐spread function and increases local color mixing within the ROI, which can bias the L*, a* and b* averages and further inflate ΔE; the resolution deficit of zoom lenses is thus not merely a parallel finding but a plausible contributor to their inferior color performance (Dugonik et al. 2020; Yang et al. 2022; Koren 2020).

Our results can be compared with Yilmaz et al. (2023), who imaged four VITA 3D‐Master tabs (*n* = 7) with a DSLR, a 100 mm macro lens and a ring flash, comparing two cross‐polarizing filters against a non‐polarizer and reporting CIEDE2000 (Δ*E*_00). They found that every group—including both cross‐polarized conditions—exceeded the CIEDE2000 acceptability threshold (Δ*E*_00 > 1.80), and that polarization did not consistently improve trueness: for the high‐value 1M1 tab, the polarizing filters produced larger errors than the non‐polarizer. At first sight, this appears to conflict with our finding of clinically acceptable macro accuracy. The discrepancy is reconciled on three grounds. First, metric: they used ΔE_00 (AT = 1.80) whereas we used ΔEab (AT = 2.70); the metrics and their thresholds are not interchangeable, so the numbers cannot be compared directly. Second, calibration: the decisive difference is our per‐session custom white balance from a gray card combined with a locked linear‐RAW, single‐profile pipeline referenced to a per‐tab spectroradiometer reading; trueness in cross‐polarized photography is highly sensitive to white balance and profiling, and their protocol differed here, with filtered images drifting notably in a*. Third, hardware: filter brand and polarizer quality alter chromatic transmission, and their filter‐dependent shifts reinforce that technique and calibration—not the mere presence of a macro lens or a polarizer—govern accuracy. Our results are consistent with their broader conclusion that photographic technique must be optimized to reach clinical acceptability, and extend it by isolating the lens‐optics contribution under a fixed, calibrated pipeline (Mohammed et al. 2026; Yilmaz et al. 2023).

Full‐frame bodies achieve lower ΔEab than APS‐C bodies, likely owing to larger photosites, better signal‐to‐noise ratio and wider dynamic range. Even so, the present macro‐vs‐zoom analysis suggests that lens selection and protocol standardization influence shade accuracy more than sensor size alone. Clinically, cross‐polarization, white‐balance calibration and a dedicated macro lens should be prioritized before investing in sensor upgrades; a full‐frame body offers an incremental advantage but cannot compensate for suboptimal optics or workflow (Jasim et al. 2024).

For practice, clinicians should use a dedicated macro lens for shade photography and avoid general‐purpose zoom lenses for this purpose. Among modern macro lenses, choice can be guided by ergonomics, working distance, and budget, since color differences are negligible under a standardized protocol. A cross‐polarized ring or twin‐flash configuration and a neutral gray card for custom white balance are essential to control specular reflection and ensure session‐to‐session consistency, and RAW capture with in‐camera and software enhancements disabled helps preserve true color (Yilmaz et al. 2023; Rashid et al. 2023; Gonzalez‐Chavez et al. 2025).

Shade tabs rather than natural teeth were used to allow rigorous standardization, avoiding the translucency, micro‐texture, and moisture effects of natural teeth; cross‐polarization aids generalizability, but in vivo validation remains warranted. A single camera body (Nikon D610) was used; although this body was held constant across all lenses and therefore cannot bias between‐lens contrasts, absolute ΔEab and MTF values may differ on other sensors and image‐processing pipelines. Moreover, all lenses tested were F‐mount designs, whereas many clinicians work with Canon (EF/RF) or Sony (E‐mount) systems. Because sensor color‐filter‐array spectral characteristics, sensor color interpolation (demosaicing) algorithms, and color rendering pipelines—including the camera profiles applied during RAW conversion—differ between manufacturers, our findings, and in particular the absolute ΔEab values, may not transfer directly to Canon or Sony bodies (Burggraaff et al. 2019). We consider the relative conclusions that are grounded in lens optics—the mutual equivalence of contemporary macro lenses and the inferiority of general‐purpose zooms at close focus—likely to be robust across platforms, since the camera body and processing pipeline were held constant for every comparison; this expectation, however, itself requires empirical verification. Within the Nikon system, the field is moving toward mirrorless bodies: although the tested F‐mount optics mount on Nikon Z bodies through an optically empty FTZ/FTZ II adapter that preserves the optical behavior studied here, Z‐series sensors and color pipelines differ from those of the D610, so absolute values may not transfer verbatim to mirrorless bodies. The zoom lenses tested were general‐purpose rather than specialized macro‐zoom designs. Because the reference was acquired at 45°/0° under D65 with the specular component included, whereas the photographs were cross‐polarized and coaxial, the absolute ΔEab values include a systematic reference‐related offset (see Discussion); between‐lens contrasts are unaffected. Finally, the referenced sensor comparison used ΔE_00 rather than ΔEab; because the two metrics are not interchangeable, cross‐study numerical comparisons should be interpreted with caution (Ruiz‐López et al. 2022; Gómez‐Polo et al. 2022).

Future work should extend to in vivo conditions, comparing macro and zoom lenses on natural teeth under controlled cross‐polarized illumination, and should validate clinical equivalence among macro lenses across multiple bodies and sensor platforms, including native Z‐mount, Canon RF and Sony E macro‐optics on current mirrorless systems, to confirm that the present findings transfer to the camera systems most widely used in clinical practice, as these become standard. Contemporary smartphone cameras paired with polarizing accessories also warrant quantitative evaluation, as does the integration of standardized, cross‐polarized protocols into routine clinical workflows to reduce remakes and improve laboratory communication.

## 5 Conclusion

Under a standardized, cross‐polarized, calibrated protocol, macro lenses from major manufacturers are clinically equivalent in color accuracy and yield high sharpness. The tested general‐purpose zoom lenses were unsuitable for shade matching under the standardized protocol. Because photographic technique was standardized rather than varied, this study demonstrates that, within a calibrated workflow, macro‐lens brand has no clinically meaningful effect; it was not designed to rank technique against brand.

## Author Contributions

**Ahmed Ali Jasim:** conceptualization (lead), methodology (lead), investigation (lead), data curation (lead), writing – original draft (lead), writing – review and editing (equal), project administration (lead). **Amer Abbas Mohammed:** formal analysis (lead), funding acquisition (lead), writing – review and editing (supporting). **Huda Ahmed Abdullah:** investigation (supporting), resources (supporting), writing – review and editing (supporting). **Samah Fadhil Al‐Qazzaz:** conceptualization (supporting), methodology (supporting), investigation (supporting), writing – review and editing (supporting). **Mafaz Mahdi Mohsin:** methodology (supporting), data curation (supporting), writing – review and editing (supporting). All authors read and approved the final version of the manuscript submitted for publication, and agree to be accountable for all aspects of the work.

## Funding

The authors have nothing to report.

## Conflicts of Interest

The authors declare no conflicts of interest.

## Data Availability

The data that support the findings of this study are available from the corresponding author upon reasonable request.

## References

[cre270484-bib-0001] Akl, M. A. , D. E. Mansour , and F. Zheng . 2023. “The Role of Intraoral Scanners in the Shade Matching Process: A Systematic Review.” Journal of Prosthodontics 32: 196–203. 10.1111/jopr.13576 35919949

[cre270484-bib-0002] Ali, S. F. M. , A. A. Jasim , and S. J. Muhsen . 2024. “Assessment of Image Quality and Child Acceptance for DSLR, Bridge and Smartphone Cameras Used in Dental Photography. (A Comparative Study).” Tikrit Journal for Dental Sciences 12: 58–68.

[cre270484-bib-0003] Binhuraib, H. , S. Aloqayli , S. Alkhalifah , et al. 2024. “Digital Shade Matching Techniques in Fixed Prosthodontics.” Journal of Healthcare Sciences 04: 34–40.

[cre270484-bib-0004] Burggraaff, O. , N. Schmidt , J. Zamorano , et al. 2019. “Standardized Spectral and Radiometric Calibration of Consumer Cameras.” Optics Express 27: 19075–19101. 10.1364/OE.27.019075 31503673

[cre270484-bib-0005] Diab, N. , K. Nour , and M. Nasser . 2024. “Effect of Enamel Thickness and Dentin Shade of Resin Composite on the Final Color Reproduction.” Ain Shams Dental Journal 33: 97–103. 10.21608/asdj.2023.224645.1182.

[cre270484-bib-0006] Dugonik, B. , A. Dugonik , M. Marovt , and M. Golob . 2020. “Image Quality Assessment of Digital Image Capturing Devices for Melanoma Detection.” Applied Sciences 10: 2876.

[cre270484-bib-0007] Farah, R. I. , A. S. Almershed , B. F. Albahli , and S. N. Al‐Haj Ali . 2022. “Effect of Ambient Lighting Conditions on Tooth Color Quantification in Cross‐Polarized Dental Photography: A Clinical Study.” Journal of Prosthetic Dentistry 128: 776–783. 10.1016/j.prosdent.2021.01.015 33640092

[cre270484-bib-0008] Gómez‐Polo, C. , M. Gómez‐Polo , N. Quispe López , M. Portillo Muñoz , and J. Montero . 2022. “3D Master Toothguide Is Adequate to Subjective Shade Selection?” Medicina 58: 457. 10.3390/medicina58030457 PMC8954102 35334632

[cre270484-bib-0009] Gonzalez‐Chavez, J. A. , U. Soto‐Barreras , B. Perez‐Aguirre , M. Nevarez‐Rascon , C. E. Villegas‐Mercado , and R. A. Dominguez‐Perez . 2025. “Reliability of Dental Shade Selection Methods: Agreement Among Spectrophotometer, Intraoral Scanner, and Cross‐Polarization Photography.” Journal of Esthetic and Restorative Dentistry 37: 1784–1790. 10.1111/jerd.13458 40070050

[cre270484-bib-0010] Gowda B N, V. K. , S. Gauni , K. Gurushankar , and V. Maik . 2022. “Application in Optical Design: Optimization for High Intensity and Aberration Free Camera Lens System.” Optik 262: 168764.

[cre270484-bib-0011] Grigollo Patussi, E. , B. C. Garcia Poltronieri , R. Ottoni , J. Bervian , C. Lisboa , and P. H. Corazza . 2019. “Comparisons Between Photographic Equipment for Dental Use: DSLR Cameras vs. Smartphones.” Revista da Faculdade de Odontologia ‐ UPF 24: 198–203.

[cre270484-bib-0012] Harikrishnan, S. , S. Dinesh , A. Sivakumar , et al. 2023. “Comparative Evaluation of Various Lens and Ring Flash Combination for Intraoral Photography.” Nigerian Journal of Clinical Practice 26: 1800–1807. 10.4103/njcp.njcp_120_23 38158345

[cre270484-bib-0013] Hasbini, O. , R. Bourgi , N. Kharouf , et al. 2025. “Digital Dentistry and Imaging: Comparing the Performance of Smartphone and Professional Cameras for Clinical Use.” Prosthesis 7: 77.

[cre270484-bib-0014] Jasim, A. A. , S. F. Al‐Qazzaz , and N. H. Radhi . 2024. “Assessment of Full Frame and Crop Frame Dslr Cameras for Dental Shade Selection: in Vitro Comparative Study.” Journal of Stomatology 77: 278–284.

[cre270484-bib-0015] Koren, N. L. 2020. “Correcting Misleading Image Quality Measurements.” Electronic Imaging 32: 242‐1.

[cre270484-bib-0016] Kupke, L. , C. Schwahn , K.‐F. Krey , and A. Ratzmann . 2025. “In Vivo Reproducibility of Tooth Shade Determination on Intraoral Scans Compared to the Dental Spectrophotometric Method.” Clinical Oral Investigations 29: 185. 10.1007/s00784-025-06240-5 PMC11910410 40088302

[cre270484-bib-0017] Mahn, E. , S. C. Tortora , B. Olate , F. Cacciuttolo , J. Kernitsky , and G. Jorquera . 2021. “Comparison of Visual Analog Shade Matching, a Digital Visual Method With a Cross‐Polarized Light Filter, and a Spectrophotometer for Dental Color Matching.” Journal of Prosthetic Dentistry 125: 511–516. 10.1016/j.prosdent.2020.02.002 32197819

[cre270484-bib-0018] Mohamed‐Ali, S. F. , A. A. Jasim , M. A. Mahmood , and M. M. Mohsin . 2024. “The Efficacy of Populus Euphratica and Myrtus Communis Herbal Mouthwash on Gingivitis in Pediatric Patients Diagnosed Clinically and With Photometric Cielab Analysis: An In Vivo Randomized Double‐Blind Clinical Study.” International Journal of Clinical Pediatric Dentistry 17: 918–924. 10.5005/jp-journals-10005-2900 PMC11451873 39372348

[cre270484-bib-0019] Mohammed, A. A. , H. A. Abdullah , M. N. Abdulghani , A. A. Jasim , S. F. Al‐Qazzaz , and M. M. Mohsin . 2026. “The Influence of Background Color on Colorimetric Accuracy in Dental Photography: A Multi‐Specimen Spectrophotometric Analysis.” Romanian Journal of Stomatology 72, no. l: 169.

[cre270484-bib-0020] Pal, S. 2013. “Aberration Correction of Zoom Lenses Using Evolutionary Programming.” Applied Optics 52: 5724. 10.1364/AO.52.005724 23938425

[cre270484-bib-0021] Paravina, R. D. , R. Ghinea , L. J. Herrera , et al. 2015. “Color Difference Thresholds in Dentistry.” Journal of Esthetic and Restorative Dentistry 27: S1–S9. 10.1111/jerd.12149 25886208

[cre270484-bib-0022] Paravina, R. D. , M. M. Pérez , and R. Ghinea . 2019. “Acceptability and Perceptibility Thresholds in Dentistry: A Comprehensive Review of Clinical and Research Applications.” Journal of Esthetic and Restorative Dentistry 31: 103–112. 10.1111/jerd.12465 30891913

[cre270484-bib-0023] Paulson, C. A. , J. J. Price , K. W. Koch , et al. 2019. “Industrial‐Grade Anti‐Reflection Coatings With Extreme Scratch Resistance.” Optics Letters 44: 5977. 10.1364/OL.44.005977 32628197

[cre270484-bib-0024] Rashid, F. , T. H. Farook , and J. Dudley . 2023. “Digital Shade Matching in Dentistry: A Systematic Review.” Dentistry Journal 11: 250. 10.3390/dj11110250 PMC10670912 37999014

[cre270484-bib-0025] Ruiz‐López, J. , M. M. Perez , C. Lucena , et al. 2022. “Visual and Instrumental Coverage Error of Two Dental Shade Guides: An In Vivo Study.” Clinical Oral Investigations 26: 5961–5968. 10.1007/s00784-022-04556-0 PMC9474559 35639206

[cre270484-bib-0026] Sajjadi, S. , B. Khosravanifard , M. Esmaeilpour , V. Rakhshan , and F. Moazzami . 2015. “The Effects of Camera Lenses and Dental Specialties on the Perception of Smile Esthetics.” Journal of Orthodontic Science 4: 97. 10.4103/2278-0203.173426 PMC4759977 26952147

[cre270484-bib-0027] Tomofuji, T. , N. Okada , S. Hiraki , and J. Nagatsuka . 2001. “A New Coating Technique for Lenses Which Have Steep Curved Surface.” In Optical Interference Coatings MD2. OSA. 10.1364/OIC.2001.MD2.

[cre270484-bib-0028] Vergöhl, M. , M. Britze , S. Bruns , A. Pflug , and V. Kirchner “Deposition of Demanding Optical Coatings on Curved Substrates.” In *Optical Interference Coatings Conference (OIC) 2019 WD.6* (OSA, Santa Ana Pueblo, New Mexico, 2019). 10.1364/OIC.2019.WD.6.

[cre270484-bib-0029] Yang, M. , G. Yin , H. Wang , J. Dong , Z. Xie , and B. Zheng . 2022. “A Underwater Sequence Image Dataset for Sharpness and Color Analysis.” Sensors 22: 3550. 10.3390/s22093550 PMC9100472 35591240

[cre270484-bib-0030] Yilmaz, B. , D. Ö. Dede , E. Diker , M. Fonseca , W. M. Johnston , and A. S. Küçükekenci . 2023. “Effect of Cross‐Polarization Filters on the Trueness of Colors Obtained With a Single‐Lens Reflex Camera, Macro Lens, and a Ring Flash.” Journal of Esthetic and Restorative Dentistry 35: 878–885. 10.1111/jerd.13053 37073977

[cre270484-bib-0031] Yung, D. , A. Tse , R. Hsung , M. G. Botelho , E. Pow , and W. Lam . 2023. “Comparison of the Colour Accuracy of a Single‐Lens Reflex Camera and a Smartphone Camera in a Clinical Context.” Journal of Dentistry 137: 104681. 10.1016/j.jdent.2023.104681 37648197

